# Fast selection of maribavir resistant cytomegalovirus in a bone marrow transplant recipient

**DOI:** 10.1186/1471-2334-13-330

**Published:** 2013-07-19

**Authors:** Axel Schubert, Karoline Ehlert, Susanne Schuler-Luettmann, Eva Gentner, Thomas Mertens, Detlef Michel

**Affiliations:** 1 Institut für Virologie, Universitätsklinikum Ulm, Albert-Einstein-Allee 11, 89081 Ulm, Germany; 2 Klinik und Poliklinik für Kinder- und Jugendmedizin, Pädiatrische Hämatologie und Onkologie, Universitätsklinikum Münster, Albert-Schweitzer-Campus 1, 48149 Münster, Germany; 3 Institut für Medizinische Mikrobiologie, Klinische Virologie, Von-Stauffenberg-Str. 36, 48151 Münster, Germany

**Keywords:** Antiviral resistance, Cytomegalovirus, Maribavir, Ganciclovir, Foscarnet, Bone marrow transplantation

## Abstract

**Background:**

Human cytomegalovirus infections are still significant causes of morbidity and mortality in transplant recipients. The use of antiviral agents is limited by toxicity and evolving resistance in immunocompromised patients with ongoing viral replication during therapy. Here, we present the first documented case of genotypic resistance against maribavir in a bone marrow transplant (BMT) recipient.

**Case presentation:**

The female 13-year-old patient was suffering from a refractory cytopenia. Ganciclovir, foscarnet, cidofovir, leflunomide and maribavir, an inhibitor of the cytomegalovirus UL97 protein, were administered to treat a therapy-resistant cytomegalovirus infection. Viral mutations conferring resistance against nucleotide and pyrophosphate analogs as well as maribavir (MBV) have evolved sequentially. Particularly, impressive was the fast emergence of multiple mutations T409M, H411Y and H411N conferring maribavir resistance after less than 6 weeks.

**Conclusion:**

We describe the fast emergence of cytomegalovirus variants with different maribavir resistance associated mutations in a bone marrow transplant recipient treated with MBV 400 mg p.o. twice per day. The results suggest that a high virus load permitted a selection of several but distinct therapy-resistant HCMV mutants. Since a phase II study with MBV is intended for the treatment of resistant or refractory HCMV infections in transplant recipients this has to be kept in mind in patients with high viral loads during therapy (NCT01611974).

## Background

All compounds approved for systemic treatment of human cytomegalovirus (HCMV) infections target the viral DNA polymerase [1-3]. Furthermore, all of these compounds are limited by dose related toxicities and the emergence of resistant virus mutants. The experimental drug maribavir (MBV) primarily inhibits the viral UL97 kinase [3-5]. MBV is orally bioavailable and was shown to have anti-HCMV activity in early clinical trials [6-8]. However, this activity could not be confirmed in a phase III prophylaxis trial involving transplant recipients who obtained 100 mg twice per day [9]. Recently, MBV was used in a salvage treatment study using a higher dosage of 400 mg twice per day in 6 cases, who had failed to respond to other therapies and/or had known ganciclovir-resistant HCMV [10]. In one of these patients, a heart transplant recipient, genotypic MBV resistance has been observed after 10 weeks of MBV treatment [10,11]. To our knowledge with the exception of this solid organ recipient no other cases of genotypic MBV resistance in treated patients have been published so far. Here, we describe the fast emergence of individual cytomegalovirus variants with different maribavir resistance associated mutations in a bone marrow transplant recipient treated with MBV 400 mg p.o. twice per day.

## Methods

Maribavir was supplied by ViroPharma and administered at a dose of 400 mg p.o. twice per day.

Genotypic resistance testing was performed by polymerase chain reaction (PCR) amplification of a 870 bp HCMV UL97 region [12] and a 2.1 kb fragment of the viral polymerase (UL54, codons 300–1000) directly from EDTA blood and urine samples. DNA sequencing of UL97 was performed allowing detection of all described mutations conferring ganciclovir resistance and most of the mutations described after in vitro selected maribavir resistance. DNA sequencing of UL54 was performed allowing detection of all described mutations conferring resistance against ganciclovir, cidofovir, and foscarnet. To determine whether multiple mutations detected in patient’s specimens occurred within the same or different viral genomes, UL97 fragments were amplified from the extracted DNA, ligated into the cloning vector pJET1.2/blunt (Fermentas, Lituania) and transfected into *E*. *coli* DH5α. The UL97 regions of the resulting clones were sequenced as described above.

## Case presentation

The bone marrow transplant recipient was a 13-year-old HCMV seropositive girl. The diagnosis of refractory cytopenia in July 2010 without genetic and molecular abnormalities was followed by bone marrow transplantation (BMT).

Before transplantation in March 2011 supportive treatment with transfusions and antibiotics was given. The non-myeloablative regimen with fludarabine (4x40 mg/m^2^), thiotepa (3x5 mg/kg KG) and anti-thymocyte globulin (3x20 mg/kg KG) was followed by transfusion of the marrow transplant from an unrelated 10/10 compatible HCMV seronegative (D-) donor. Neutrophil engraftment occurred very late at day +43.

**Figure 1:**
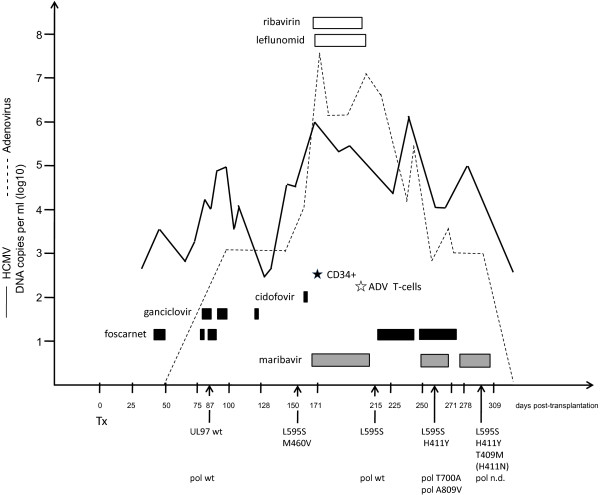
**Time course of antiviral treatments and events.** The viral loads of adenovirus (dashed) and HCMV are depicted as DNA copies per ml EDTA blood. The durations of treatments with ganciclovir, foscarnet and cidofovir are shown as black boxes and with maribavir as grey boxes, respectively. Ribavirin and leflunomide are presented as blank boxes. The boost with selected CD34+ cells is depicted as black star. The administration of adenovirus-specific T-cells is illustrated by a white star. Tx, transplantation; UL97, HCMV UL97 kinase; wt, wild type; pol, HCMV polymerase (UL54). Mutations detected by direct genotyping from the specimens are shown in the single letter code (L592S, lysine at amino acid 595 to serine). The mutation H411N (brackets) was detected by cloning but not by direct genotyping. ADV, adenovirus; n.d., not detected.

After detection of an active HCMV infection at week six post transplantation, foscarnet was administered because of lower hematotoxic side effects (see Figure 1). However, after one week this therapy had to be discontinued because of ulcera in the genital and in the oral mucosa. Due to the ongoing viral replication, at week 10, a therapy with foscarnet (180 mg/kg KG) and ganciclovir (10 mg/kg KG) was initiated for 3 weeks, resulting in a considerable decrease of the viral load in the blood. However, during this period the girl developed lung infiltrates consistent with the diagnosis of viral induced pneumonia. At this time, UL97 and UL54 genotyping revealed wild-type sequences. The virus carried the UL97 polymorphism D605E as a genetic marker, which is not associated with ganciclovir resistance [13].

Foscarnet therapy was again discontinued at week 13 due to adverse side effects. After onset of an acute graft-versus-host-disease (GvHD), followed by an intensified immunosuppressive therapy, at week 25 a stem cell boost with selected CD34-positive peripheral blood stem cells became necessary, because of a secondary graft failure. During this period a substantial increase of the HCMV viral load up to more than 10^6^ genome equivalents per ml (ge/ml) in EDTA blood occurred. UL97 genotyping revealed the emergence of HCMV variants with mutations, M460V and L595S, conferring GCV resistance. Additionally, due to an active adenovirus (species A) infection in August 2011 cidofovir and ribavirin were administered.

Since one cycle of cidofovir (5 mg/kg KG) did not result in a significant reduction of the HCMV viral load, a salvage therapy was induced which consisted of leflunomide (80 mg p.o. initially for the first two days, followed by 20 mg once per day) and maribavir (starting dose of MBV was 400 mg p.o. twice per day). The duration of treatment over five weeks resulted in an improvement and the patient experienced a temporary decline of the HCMV viral load to approximately 10^4^ ge/ml. The virus load remained at that level.

A transfusion of adenovirus-specific donor-derived T-cells was performed in October (week 29). One week after termination of the salvage therapy, UL97 genotyping revealed that the M460V variant was no longer detectable. However, the L595S mutation was still present. Since the UL54 genotyping exhibited no resistance-associated mutation, foscarnet therapy was again initiated at week 32. At week 35 an increase in the virus load was observed associated with a relapse of pneumonitis. Therefore, a new adjustment of therapy with MBV (400 mg p.o. twice per day) was initiated. Two weeks later genotyping revealed a compilation of different UL97 and UL54 mutations. The UL54 sequences showed a mixture of T700A and A809V conferring potential low resistance to GCV and resistance to foscarnet, respectively [14]. In addition to the already known mutation L595S, a new mutation was detected in the UL97 region, namely the mutation H411Y which has been described to confer resistance to maribavir [15]. However, the combination therapy with foscarnet and MBV was continued due the low proportion of MBV resistant virus in the patient and the lack of alternative compounds.

At week 40 foscarnet was withdrawn. After two further weeks of MBV administration, UL97 genotyping exhibited an increase of the virus population carrying the mutation H411Y and further more, the emergence of a second mutation T409M also conferring MBV resistance [15].

In order to solve the question whether the detected mixture of mutations consisted of different single, double or multiple HCMV mutants, UL97 amplicons were cloned into *E.coli* and individual clones were further analyzed. Sequencing revealed that all clones (n=33) carried the UL97 marker polymorphism D605E. Most of the clones carried wild type UL97 sequences (67%). However, certain clones carried one of the mutations L592S, H411Y, or T409M. As already reported by Strasfeld et al. [11] in a solid organ recipient, none of the clones contained more than one single resistance mutation which indicates that the mutations were located on separate viral genomes. Surprisingly, one clone exhibited the mutation H411N also conferring MBV resistance which had not been detected by the direct sequencing of UL97 from the patient’s specimens. Regrettable, despite several attempts no virus isolates could be obtained.

Fortunately, the patient recovered from the HCMV and adenovirus infection. Until today she did not experience further serious virus associated events and can attend school.

## Discussion

In this study we present data that maribavir resistance occurred relatively rapid in a bone marrow transplant (BMT) recipient after less than 6 weeks of MBV treatment. Previous MBV trials included mainly short durations of treatment of up to four weeks or prophylactic use of up to 12 weeks in patients without active infection at the onset [6-9]. Therefore, in these studies drug resistance was not detected.

Avery and coworkers presented data from six patients (five solid organ transplant recipients and one hematopoietic stem cell transplant recipient) who had failed to respond to other therapies and/or carried a known ganciclovir-resistant HCMV [10]. The starting dose of MBV was 400 mg p.o. twice per day and was increased up to 800 mg twice per day in one patient. Patients were treated for a median of 207 days (range, 15 to 376). Four of the 6 patients had no detectable HCMV DNAemia within 6 weeks after starting MBV therapy. One solid organ recipient, who had an initial high viral load, developed a MBV resistance mutation.

In our patient, MBV resistance was associated with an initial high viral load that could not be cleared with ganciclovir, cidofovir, foscarnet, lefunomide or maribavir. The patient developed genotypic MBV resistance after a second short period of therapy. This is the first documented case of genotypic resistance against this drug in a bone marrow transplant recipient. Probably, the high virus load permitted the faster selection of several, individual UL97 mutants shortly after readministration of MBV.

In vitro data show that UL97 mutations V353A, L397R, T409M, and H411L/N/Y, are all located near the kinase ATP binding domain, and confer moderate to high levels of MBV resistance without GCV cross-resistance [3,5,15]. The mutations T409M, and H411Y found in our patient after direct sequencing belong to the most common mutations induced in vitro and confer a ~9-fold to ~200-fold increase in IC_50_ values [5,15]. Surprisingly, several independent mutated virus variants have been identified in our patient. After analysis of cloned UL97 amplicons, none of the clones contained more than one single mutation. This indicates that several mutated virus variants were selected separately during treatment which is in agreement with data from Strasfeld et al. [11]. Furthermore, by detection of the H411N mutation after sequencing separate *E. coli* clones we could show that individual HCMV variants might be present in the patient being beyond the detection limit of the routinely performed direct genotyping from patient specimens.

Notably, in vitro results suggest that the GCV-resistance mutations M460V/I confer MBV hypersensitivity [5]. These findings might be supported by our results in vivo. After the onset of the maribavir therapy, the M460V mutation disappeared but not the L594S.

Although UL97 deficient strains are highly impaired in their replication capacity, it remains questionable whether MBV should be administered as salvage therapy to immunosuppressed patients with a high viral load at the beginning and during therapy at all.

In conclusion, therapy adjustment in immunosuppressed patient with high virus loads over longer periods should only be done after HCMV genotyping. This holds true particularly for the changing from a usually effective antiviral drug with fewer side effects (e.g. GCV) to antiviral drugs with less efficiency or higher toxicity (e.g. cidofovir or foscarnet, respectively) without any evidence of viral drug (genotypic or phenotypic) resistance. Furthermore, due to short time drug changes different HCMV mutants might be selected from the virus population.

## Conclusion

Our case suggests that a high virus load permitted fast selection of several, individual UL97 mutants and may contribute to a faster occurrence of overall (multi-) drug resistance. Since a phase II study with MBV is intended for the treatment of resistant HCMV or HCMV infections after treatment failure in transplant recipients this has to be kept in mind in patients with a high viral load at the beginning or during antiviral therapy (NCT01611974) [16].

## Consent

The patient was treated with standard patient care and the parent consented to publication. Written informed consent was obtained from the patient for publication of this case. A copy of the written consent is available for review by the Editor-in-Chief of this journal.

## Abbreviations

HCMV: Human cytomegalovirus; MBV: Maribavir; PCR: Polymerase chain reaction; UL97: Unique long open reading frame 97; UL54: Unique long open reading frame 54; BMT: Bone marrow transplantation; GvHD: Graft-versus-host-disease.

## Competing interests

D.M. is consultant for virological diagnosis for AiCuris GmbHCo.KG. The authors have received no funding for the preparation of this manuscript. The authors declare that they have no conflicts of interests. The information has not been presented on any meeting.

## Authors’ contributions

AS, KE, SSL, EG, TM and DM have been involved in acquisition and interpretation of data. KE has been involved in patient clinical care and reviewing medical records. AS, SSL and DM carried out the laboratory assays and contributed to analysis. AS, EG, and DM contributed to molecular studies. AS, TM, and DM drafted the manuscript. TM reviewed and revised this paper, and gave final approval to submit for publication. All authors read and approved the final manuscript.

## Pre-publication history

The pre-publication history for this paper can be accessed here:

http://www.biomedcentral.com/1471-2334/13/330/prepub
